# Ventral Tegmental Area Dopamine Neural Activity Switches Simultaneously with Rule Representations in the Medial Prefrontal Cortex and Hippocampus

**DOI:** 10.1523/JNEUROSCI.1670-24.2025

**Published:** 2025-03-17

**Authors:** Mingxin Ding, Porter L. Tomsick, Ryan A. Young, Shantanu P. Jadhav

**Affiliations:** ^1^Graduate Program in Neuroscience, Brandeis University, Waltham, Massachusetts 02453; ^2^Undergraduate Program in Neuroscience, Brandeis University, Waltham, Massachusetts 02453; ^3^Department of Neuroscience, Virginia Polytechnic Institute and State University, Blacksburg, Virginia 24061; ^4^Department of Psychology, Brandeis University, Waltham, Massachusetts 02453; ^5^Volen National Center for Complex Systems, Brandeis University, Waltham, Massachusetts 02453

**Keywords:** dopamine, hippocampal CA1, medial prefrontal cortex, rule switching, VTA

## Abstract

Multiple brain regions need to coordinate activity to support cognitive flexibility and behavioral adaptation. Neural activity in both the hippocampus (HPC) and medial prefrontal cortex (mPFC) is known to represent the spatial context and is sensitive to reward and rule alterations. Midbrain dopamine (DA) activity is key to reward-seeking behavior and learning. There is abundant evidence that midbrain DA modulates HPC and PFC activity. However, it remains underexplored how these networks engage dynamically and coordinate temporally when animals adjust their behavior according to changing reward contingencies. In particular, is there any relationship between DA reward prediction change during rule switching and rule representation changes in mPFC and CA1? We addressed these questions using simultaneous recordings of neuronal population activity from the hippocampal area CA1, mPFC, and ventral tegmental area (VTA) in male TH-Cre rats performing two spatial working memory tasks with frequent rule switches in blocks of trials. CA1 and mPFC ensembles showed rule-specific activity both during maze running and at reward locations, with mPFC rule coding more consistent across animals compared with CA1. Optogenetically tagged VTA DA neuron firing activity responded to and predicted reward outcome. We found that the correct prediction in DA emerged gradually over trials after rule switching in coordination with transitions in mPFC and CA1 ensemble representations of the current rule after a rule switch, followed by behavioral adaptation to the correct rule sequence. Therefore, our study demonstrates a crucial temporal coordination between the rule representation in mPFC/CA1, the dopamine reward signal, and behavioral strategy.

## Significance Statement

This study examines neural activity in mammalian brain networks that support the ability to respond flexibly to changing contexts. We use a rule-switching spatial task to examine whether the key reward-responsive and predictive dopamine (DA) activity changes in coordination with changes in rule representations in key cognitive regions, the medial prefrontal cortex (mPFC), and hippocampus. We first established distinct rule representations in mPFC and hippocampus and predictive coding of reward outcomes by DA neuronal activity. We show that the DA reward prediction after a rule switch develops in temporal coordination with changes in rule representations in mPFC, eventually leading to behavioral changes. These results thus provide an integrated understanding of reward prediction, cognitive representations of rules, and behavioral adaptation.

## Introduction

Behavioral flexibility is critical to survival and for adapting to a changing environment. These functions are frequently driven by changing reward conditions and supported by distributed network processing in the brain to evaluate context and respond appropriately. The hippocampal cognitive map represents spatial context (O’Keefe and Dostrovsky, 1971; O’Keefe, 1978), and hippocampal neuronal activity can also be modulated by rewards and goals (Lee et al., 2012; Gauthier and Tank, 2018; Krishnan et al., 2022). The prefrontal cortex (PFC) serves complementary cognitive functions including working memory, executive control, and context representation (Ragozzino and Kesner, 1998; Yoon et al., 2008; Horst and Laubach, 2009; Durstewitz et al., 2010; Hyman et al., 2012; Urban et al., 2014; Ma et al., 2016). The hippocampus (HPC) and prefrontal cortex have been demonstrated to coordinate temporally for adaptation of behavioral strategy when experiencing environmental/contextual changes (Guise and Shapiro, 2017; Hasz and Redish, 2020). However, much remains unexplored about how reward signals, acting as major feedback to animals’ state/action choices, coordinate with this navigation and memory system for cognitive flexibility and behavioral adaptation. The dopamine (DA) signal from the midbrain is known to be involved in reward prediction error (RPE) processing (Schultz et al., 1993, 1997) and value estimates (Roesch et al., 2007; Howe et al., 2013; Hamid et al., 2016; Dabney et al., 2020) and is causally linked to learning (Steinberg et al., 2013; Hamid et al., 2016). DA release profile and firing activity have also been reported to ramp up during reward approach and scale positively with reward quantity and probability (Howe et al., 2013; Engelhard et al., 2019; Krausz et al., 2023). How this DA signal changes during rule switches to support behavioral flexibility at short time scales of a few tens of trials over which behavioral change is observed remains less explored. In this study, we were interested in examining the dynamics of rule and context representations in the hippocampal (area CA1) and prefrontal regions during reward-guided behavioral adaptation in response to rule switching, and whether these dynamics are related to ventral tegmental area (VTA) DA neuronal activity. The primary hypotheses we aimed to test are (1) whether changes in reward-associated DA neuronal firing activity are temporally coordinated with changes in rule representations in these cognitive regions; (2) whether neuronal dynamics reflects strategy shifts over a few trials due to rule switching, thus potentially supporting a role in cognitive flexibility. The PFC receives input from and sends output to midbrain DA neurons (Carr and Sesack, 2000; Beier et al., 2015; Kabanova et al., 2015; Morales and Margolis, 2017), and PFC and DA are known to contribute to reinforcement learning in a complimentary manner, with prefrontal signals encoding predictive values and dopamine encoding prediction errors (Lak et al., 2020). In addition, DA projections to the dorsal hippocampus have been reported (Gasbarri et al., 1994), and CA1 place fields have been shown to change stability based on reward conditions and DA input (Martig and Mizumori, 2011; McNamara et al., 2014; Krishnan et al., 2022). It remains unknown if and how DA signal changes coordinate with rule and context representation in the CA1 and/or PFC and the time scale of such coordination if it exists.

To investigate these questions, we implemented a novel rule-switching spatial task for rats in a W/M maze and recorded activity simultaneously from CA1, mPFC, and VTA ensembles as animals performed this task. We found single-cell and ensemble codes of underlying rules in both the CA1 and mPFC regions. VTA reward signaling and predictive coding of reward outcomes during maze running for the two rules were confirmed. We found that this rule-specific predictive feature of DA spiking activity develops together with correct mPFC and CA1 rule decoding a few trials after a rule switch, followed by a change of behavioral strategy within a few trials. Together, this work establishes that the dynamics of DA spiking activity and mPFC and CA1 rule representation changes occur in a coordinated manner during a rule-switching task.

## Materials and Methods

### Animals and experimental design

Four adult male TH-Cre rats (450–600 g, 3–7 months, RRID: RRRC_00659; Witten et al., 2011) were used in the current study for behavior and physiology data. All procedures were conducted under the guidelines of the US National Institutes of Health and approved by the Institutional Animal Care and Use Committee at Brandeis University. Animals were bred in house, kept under a 12 h light/dark schedule, with *ad libitum* food and water access till at least 10 weeks old prior to behavioral training and experiments. After daily handling and habituation to a sleep box (30 cm long, 30 cm wide, 50 cm tall), animals were moderately food deprived to 85–90% of their initial weight to motivate reward-seeking behavior on an elevated linear track. Once reaching the criterion of obtaining 60 condensed milk rewards in a 20 min session, animals were allowed free access to food for at least a week before surgery. During surgery, virus AAV5-EF1a-DIO-hChR2(E123T/T159C)-mCherry was injected into VTA, and a custom multitetrode microdrive with optrodes was implanted targeting CA1, mPFC, and VTA (see below, Surgical procedures). Tetrodes and optrodes were gradually lowered into the target regions in the time span of 3–4 weeks to allow virus expression while animals recovered and retrained on the linear track. Recordings were performed during the process of learning and rule switching on the W track (see below, Behavioral paradigm). We included male rats only for this study due to technical limitations and animal welfare concerns. The weight of our microdrive implant can be burdensome to female rats, which are typically 250–350 g as adults, in comparison with 500–600 g for male rats. Especially for lengthy recording days (4–6 h) with at least 3–4 rule switches, it is more likely for male rats to meet the task requirements with less fatigue.

### Apparatus

The experimental setup has been previously described (Shin et al., 2023). The linear track and W track were made of aluminum sheets and painted in matte black color. For each training/recording day, the track in use was situated in a dimly lit room with distinct visual features on each side (black curtain, black/white wall, equipment table). Customized reward wells were attached to the end of each track arm and connected to the recording and control system. SpikeGadgets (San Francisco, CA) hardware—an environmental control unit (ECU) that was synchronized with physiology acquisition hardware—and custom Statescript software were used for behavior recording and automated reward dispensary. Choice correctness was determined based on the current maze rule, by the software that tracked well-visit sequences. Upon an animal's arrival at the reward port after a correct choice, the animal's nose poke was detected by an infrared beam break sensor, immediately and automatically triggering the delivery of 0.15 ml of evaporated milk from a calibrated pump. Change of rule was implemented by software control, without any human interactions or changes in the environment. Nose poke and reward delivery times were recorded together with camera frames by ECU and synchronized with electrophysiological signals (see below, Data acquisition and processing).

### Behavioral paradigm

**Figure 1.**
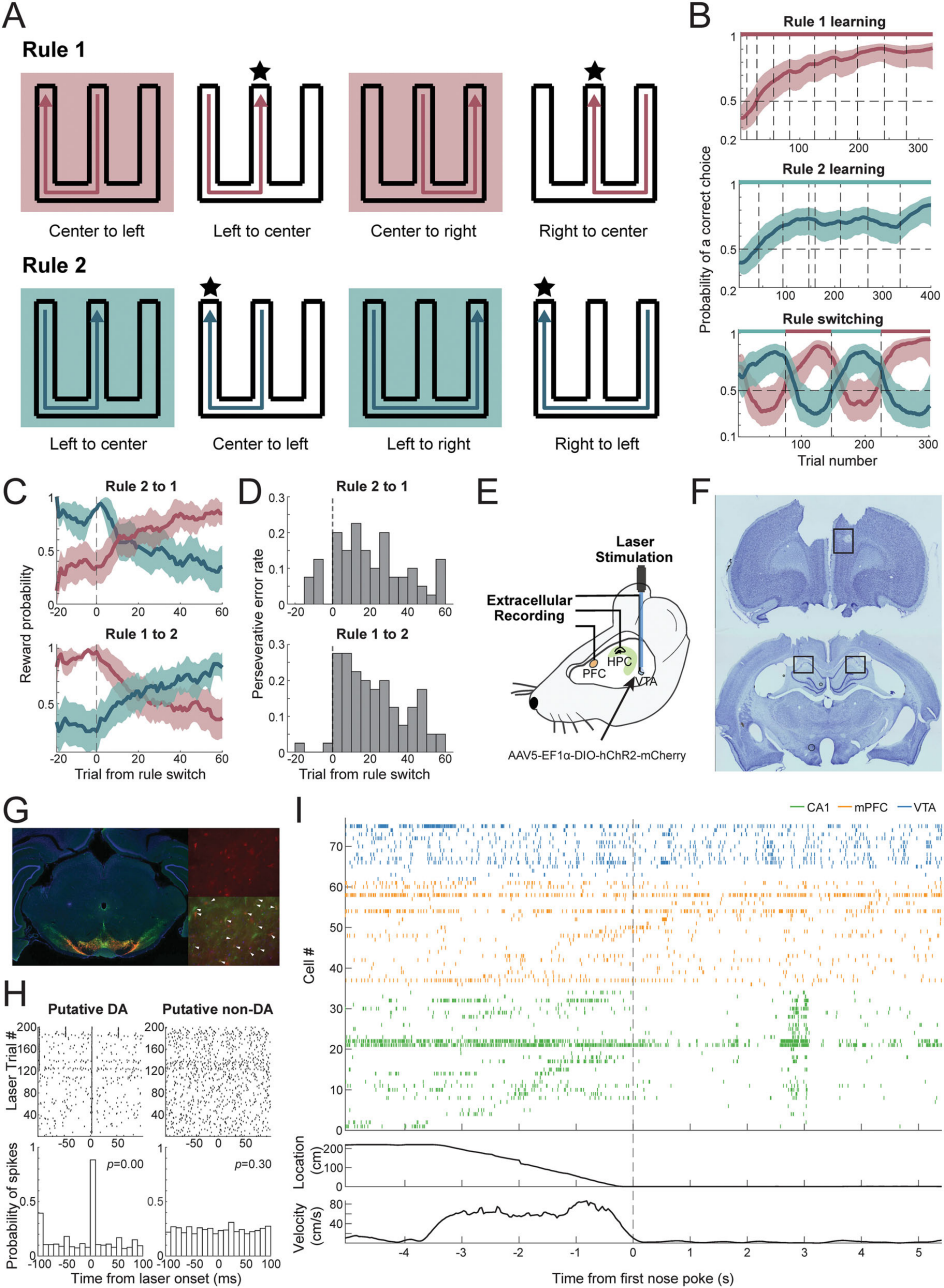
Behavior and recording paradigm. ***A***, W-track rule-switching behavior. Top, Sequence of Rule 1, left-center-right-center. Bottom, Sequence of Rule 2, center-left-right-left. Stars denote the home arm in the given rule sequence. Trajectories in shaded boxes are outbound and require working memory. The common trajectories across the two rules are left to center and center to left, with different memory demands for the rules. ***B***, Example performance curve of one animal (estimated mean ± 95% CI). Left, Rule 1 learning across behavior epochs/sessions. Middle, Rule 2 learning. Right, Rapid switching between the two rules within a single day. Note that the rule switch is not indicated by any external cue. The bar on top indicates the current rule (dark red for Rule 1, teal for Rule 2). Dashed lines in all three panels indicate epoch changes. ***C***, Average reward rate of behavioral choices based on each rule's reward contingency, for Rule 2 to 1 and Rule 1 to 2 switches separately (*n* = 8 each), aligned to rule switch trials. ***D***, Probability of perseverative error aligned to rule switch trials, for Rule 2 to 1 and Rule 1 to 2, respectively. ***E***, Recording setup. Simultaneous recording in dCA1, mPFC, and VTA regions during rule-switching behavior, with phototagging of TH+ neurons in the VTA in TH-Cre animals. ***F***, Example of Nissl-stained mPFC and CA1 slices. Rectangles indicate regions of lesion marks. ***G***, Histology in VTA. Red, virus expression of AAV5-EF1a-DIO-hChR2(E123T/T159C)-mCherry; green, antibody staining of TH+ cells; blue, DAPI. White triangles show an overlap between virus expression and antibody staining. ***H***, VTA spiking responses to laser stimulation at 0 ms. Top, Raster plot showing individual spikes aligned to each stimulation onset. Bottom, Probability of spiking. Left, Example of an optotagged neuron (*p* = 0). Right, Example of a nontagged VTA neuron [*p* = 0.30, stimulus-associated spike latency test (SALT)]. ***I***, Top, Spike raster during an example trial starting with the animal leaving the last reward location, running a trajectory to the next reward, followed by immobility and consumption at the new reward location. Green, CA1 cells; orange, mPFC; blue, VTA. Middle, Linear distance to reference reward location. Bottom, Movement speed. Dashed line: time of first nose poke at the destination reward location.

Postrecovery animals learned to perform a rule-switching task on the W track (80 cm long of each arm, 7 cm wide, Fig. 1*A*). The task consists of two rules with similar structure but different sequence assignments. The first rule is a standard W-track alternation task: animals must travel to the center arm (home) to obtain a milk reward when they set out from a side arm (inbound trajectories), whereas they need to alternate between the left and right arms when they start from the center (outbound trajectories; Jadhav et al., 2012, 2016; Maharjan et al., 2018; Shin et al., 2019). The second rule presents altered identities between the left and center arms. Therefore, the left arm becomes the new home, and animals must alternate between the center and right arms for outbound trajectories. Once animals learned both rules, they were subject to rule switching in blocks if the performance of the current rule reached 80% correct. Rule switching happened without external cues, requiring the animals to use only reward feedback to deduce the change and switch to the optimal strategy for the new rule. During a recording day, animals ran 3–4 epochs of 20–25 min duration. The run epochs were interleaved by 20–40 min sleep epochs. It typically took animals 6–12 running epochs to learn each rule and an additional training of at least 3–4 d before they could switch rapidly between rules (3–4 switches/rule blocks per day).

### Surgical procedures

Each rat received virus injection and microdrive implantation during the surgery. Microdrive fabrication and implantation procedures were similar to previous reports (Jadhav et al., 2012, 2016; Tang et al., 2017; Shin et al., 2019). Anesthesia was induced by ketamine, xylazine, and atropine cocktail and maintained by 0.5–2% isoflurane. Moreover, 500 nl of AAV5-EF1a-DIO-hChR2(E123T/T159C)-mCherry was injected into VTA bilaterally (AP, −5.6 mm; ML, ±1.0 mm; DV, −7.8 mm). Twenty-four tetrodes each targeting dorsal CA1 (AP, −3.6 to −4.0 mm; ML, ±2.2 mm; DV, −2.5 mm) and mPFC (AP, +3.0 mm; ML, ±0.9 mm; DV, −2.5 to −3.0 mm) and two optrodes targeting VTA, with each optical fiber surrounded by eight tetrodes, were encased in a microdrive and implanted against the surface (for CA1 and mPFC) or 2 mm into the brain (for VTA). Animals received postoperative analgesia and were monitored closely for at least a week before food restriction and behavioral experiments.

### Data acquisition and processing

Upon approaching target regions, CA1 was identified by the characteristic EEG features, including sharp-wave ripples and theta modulation. mPFC depth was targeted to the anterior cingulate cortex (ACC) and prelimbic (PrL) regions. VTA was identified by finding optogenetically tagged cells (see below, Cell type identification). Electrophysiological data were recorded at 30 kHz using Trodes through a 256-channel headstage (SpikeGadgets). Digital input/output (DIO) signals of nose pokes and reward delivery were simultaneously recorded. Video recordings of animal behavior were captured at 30 fps with synced timestamps to the neural and DIO recordings. All tetrodes were grounded to a screw above the cerebellum. Tetrodes of each brain region were referenced using the group average. Spiking data were bandpass filtered between 600 Hz and 6 kHz, and local field potential data were bandpass filtered between 0.5 and 400 Hz and downsampled to 1.5 kHz. Animal's position was tracked using the camera module (SpikeGadgets) to identify the red/green LEDs attached to the headstage and verified by the researcher.

Spikes were clustered using MountainSort4 (Chung et al., 2017) and followed by manual inspection and curation in MountainView. Clusters with isolation score >0.9, noise overlap <0.05, and peak signal-to-noise ratio >2 were accepted and included in this study.

### Histology

After recording, animals were put under anesthesia, and recording sites were lesioned by passing a current of 30 µA through the electrode tips. Animals were then perfused 1–2 d later using 4% formaldehyde, and the brains were kept in 4% formaldehyde and 30% sucrose solution until being sliced into 50 µm sections. The VTA slices were immunostained for tyrosine hydroxylase (TH) and imaged to verify the colocalization of virus expression and anti-TH antibody at optrode implantation sites. The CA1 and mPFC slices were stained with cresyl violet and imaged to verify tetrode locations. Our histology results indicated the span of prefrontal recording sites included the ACC (cg1) and PrL region from bregma +3.0 to +2.5 mm (Fig. 1*F*). Despite the long-standing inconsistency in naming of rodent prefrontal cortex (Uylings et al., 2003; Vertes, 2006; Laubach et al., 2018; van Heukelum et al., 2020), we felt it was most appropriate to use the term medial prefrontal cortex (“mPFC”).

### Cell type identification

Putative DA neurons were identified using optogenetic tagging at least 3 weeks after surgery to allow virus expression. In the first or last sleep epoch of each recording day, a 473 nm light was delivered to the VTA as 5 or 10 ms pulse trains at 1, 4, 10, 20, and 40 Hz to identify TH+ cells expressing ChR2, similar to previous studies (Cohen et al., 2012; Mohebi et al., 2019; Kim et al., 2020). The power of the laser was calibrated to lie within the range of 5–20 mW/mm^2^ at the tip of the fiber to avoid spike waveform distortion. The light-evoked spike latency was then tested using the stimulus-associated spike latency test (SALT; Kvitsiani et al., 2013). Units with *p* < 0.001 were classified as putative DA neurons.

CA1 and mPFC cells were separated into putative pyramidal neurons and interneurons using *k*-means clustering with parameters including spike width, peak asymmetry, and mean firing rate (Barthó et al., 2004; Sirota et al., 2008; Shin and Jadhav, 2024).

### Data analysis

#### Linearization and normalization

Each trial was defined as the time starting from leaving the last reward well, running on track, and arriving at the current reward well, until the end of the stay at the current reward well. There are six trajectories in total defined across the two rules. Occupancy of positions of each trajectory was binned into 40 equally sized spatial bins (5–6 cm/bin) on the track during running and then binned for 100 ms temporal bins at the reward wells. The occupancy was then smoothed by a five-bin-wide Gaussian kernel. The firing activity of each cell is binned and smoothed in the same fashion, and firing rates are normalized by occupancy. For dimension reduction and population activity analyses, firing rates were *z*-scored for each cell across all task epochs.

#### Behavior analysis

Behavioral performance of each animal on each recording day was estimated using a previously described state space model (Smith et al., 2004; Kim and Frank, 2009). The average reward rate was summarized for Rule 1 to 2 and Rule 2 to 1, respectively. For analysis separating performance stages, quantile ranges were set individually for each recording day to have an equal number of trials for each performance category (Figs. 3*F*, 4*F*). Behavior strategy was estimated by generating the trajectory transition matrix in a 20-trial block with a 1-trial sliding window. Pearson's correlation between each transition matrix and the optimal strategy transition matrix of each rule was computed to determine which rule the animals are currently following (Figs. 3*D*, 5*D*).

#### Spatial information

Spatial information was calculated according to Skaggs et al. (1992) to estimate the amount of spatial content of each cell's spiking activity:

$$I=\int_{x}\lambda (x)\;\log_{2}\frac{\lambda (x)}{\lambda }\;p(x)dx,$$

where *I* is the spatial information measure in bits/second, *x* is the spatial location of the animal, *p*(*x*) is the probability of the animal residing at location *x*, *λ*(*x*) is the mean firing rate of the cell at location *x*, and *λ* is the occupancy-weighted overall mean firing rate of the cell.

#### Dimensionality reduction with consistent embeddings of high-dimensional recordings using auxiliary variables

We used a newly developed nonlinear dimensionality reduction method, consistent embeddings of high-dimensional recordings using auxiliary variables (CEBRA), to uncover consistent and interpretable latent space of neural spiking data conditioned by behavioral variables (Schneider et al., 2023). Rewarded trials with performance better than the 60th percentile of the day were selected for training in CEBRA (Fig. 3*B*,*C*). Twenty percent of these trials with good performance and all trials with worse performance were left out for testing. The training trials were balanced for trajectory identities and rules. We used supervised CEBRA behavior models for both single- and multianimal training. Training labels included spatial–temporal bin, trajectory, and rule. Spiking data from each brain region were trained separately for individual animals. Parameters for CEBRA training were largely coherent with the example implementation using rat hippocampus data in Schneider et al. (2023), except for higher batch size and a larger number of iterations to improve model performance:

model_architecture = ‘offset10-model’,

time_offsets = 10,

batch_size = 2048,

learning_rate = 5e-4,

temperature = 1,

output_dimension = 3,

max_iterations = 12000,

distance = ‘cosine’,

conditional = ‘time_delta’,

num_hidden_units = 64

The whole dataset was then transformed into 3D embeddings using the trained models. To reveal common features across animals, we took advantage of the multi-animal training from CEBRA. This method allows different dimensions (numbers of cells recorded) from different sessions/animals as inputs and maintains the label space across models. Therefore, the resulting embeddings across animals are directly comparable. For multi-animal training, trial selection, model parameters (except for batch_size = 4096), and subsequent transforming process were held the same as in single-session model training.

### Rule decoding

A *k*-nearest neighbor (kNN) decoder was trained using the 3D embeddings of training data and used to decode the underlying rule for the testing data. Decoding performance of each trial was calculated as the ratio of bins with correct rule assignment (Fig. 3*D*). The decoding accuracy curve was then smoothed using a Gaussian kernel. After a rule switch, a change point was identified as the trial from which the decoding probability of the current rule was consistently higher than 50%. This process is done for embeddings from each brain region. We categorized the behavioral performance into low, mid, and high by splitting the estimated performance into one-third of the trials for each day and reported the proportion of trials with correct rule decoding in Figure 3*F*. To better estimate the timing of rule representation transitions, we used fivefold validation for training CEBRA models and decoding, resulting in five estimates for each animal and each brain region, reflected in Figure 3, *E*, *H*, and *I*.

#### DA neuron firing rate analyses

Reward responsiveness is defined as showing differences (*p* < 0.05) of firing rates at 200–1,200 ms after the first nose poke between rewarded and unrewarded trials using the Wilcoxon rank sum test. For firing rate comparisons across conditions in Figure 4*C*, firing rates are compared using the Wilcoxon signed-rank test for each spatial/temporal bin and Bonferroni-corrected for multicomparison.

The influence of behavioral factors on each DA cell's single-trial firing rates was estimated using multiple linear regression.

y=Xβ+ε

***y*** is the mean firing rate of each trial during either running or the first 3 s of the outcome period. ***X*** is the estimated behavioral factors for each trial, which included reward, binary outcome, rewarded or unrewarded; reward rate, an estimate of mean reward rate using the last five trials’ outcome; and trajectory reward rate, an estimated reward rate of a certain trajectory, using outcomes of the last five times that the animal took the same trajectory. This process is repeated for all DA neurons (*n* = 20), for both the running and reward outcome firing rates. The resulting significant coefficient values and the proportions of significant coefficients were summarized in Figure 4*H*.

## Results

We recorded neuronal activity simultaneously from the dCA1, mPFC (anterior cingulate cortex and prelimbic regions), and VTA in adult male Th-Cre rats (*n* = 4) using tetrodes in mPFC and HPC and optical fibers surrounded by tetrodes in VTA (Fig. 1*E–G*; see Materials and Methods), while animals performed a noncued spatial rule-switching task (Fig. 1*A*). Both rules in the switching task consist of two types of trajectories with different memory demands: inbound trajectories that are rewarding at the home location regardless of the animals’ travel history; and outbound trajectories that only reward animals when they choose the different arm from the last nonhome visit, requiring working memory. For Rule 1 (standard W/M maze alternation task), the middle arm is the home location and animals alternate between left and right arms for outbound trajectories. For Rule 2, the home location is switched to the left arm, and the middle and right arms become outbound destinations. Animals learned the two rules and were subsequently trained to switch between the two rules with solely the feedback of reward outcomes, and no external cues signaling the rule switch (Fig. 1*B*). Animals were first trained on Rule 1 and subsequently on Rule 2. After 7–10 d of learning and training, animals could switch between the rules 3–4 times within a recording day. Rule switching was triggered manually during a session after a threshold performance (>80% correct) was achieved on the current rule. Upon unexpected trial outcomes, animals adapted their behavioral choices quickly to obtain more rewards, evident in decreasing numbers of perseverative errors over time (Fig. 1*D*). It took 17 trials on average to reflect a Rule 2 to 1 switch in behavior and 22 trials for Rule 1 to 2 (Fig. 1*C*). The Rule 1 to 2 direction was typically more difficult for animals to achieve due to spatial asymmetry of Rule 2. During each day of recording, animals ran the tasks in three to four 20–30 min sessions, which were interleaved by 20–40 min sleep sessions. In the first or last sleep session, optotagging was performed to identify putative DA neurons in VTA (Fig. 1*H*). An example raster during one trial is shown in Figure 1*I*. Position and speed plots show animal motion and immobility at the destination reward well, and raster plots show corresponding spiking patterns in the three regions. Note that CA1 and mPFC firing activity spans the spatial locations on the track. A total of 143 CA1 neurons, 128 mPFC neurons, and 53 VTA neurons (20 TH+ neurons) from four animals, one recording day for each, were included in this study (Table 1).

**Table 1.** Clustered cell counts by brain region and cell type

Animal	CA1 Pyr	CA1 Int	mPFC Pyr	mPFC Int	VTA DA	VTA non-DA
TH105	34	7	26	4	4	7
TH212	14	5	34	5	8	11
TH510	44	5	30	2	3	6
TH605	31	3	25	2	5	9
Total	123	20	115	13	20	33

### CA1 and mPFC neurons show differentiated activity across rules

**Figure 2.**
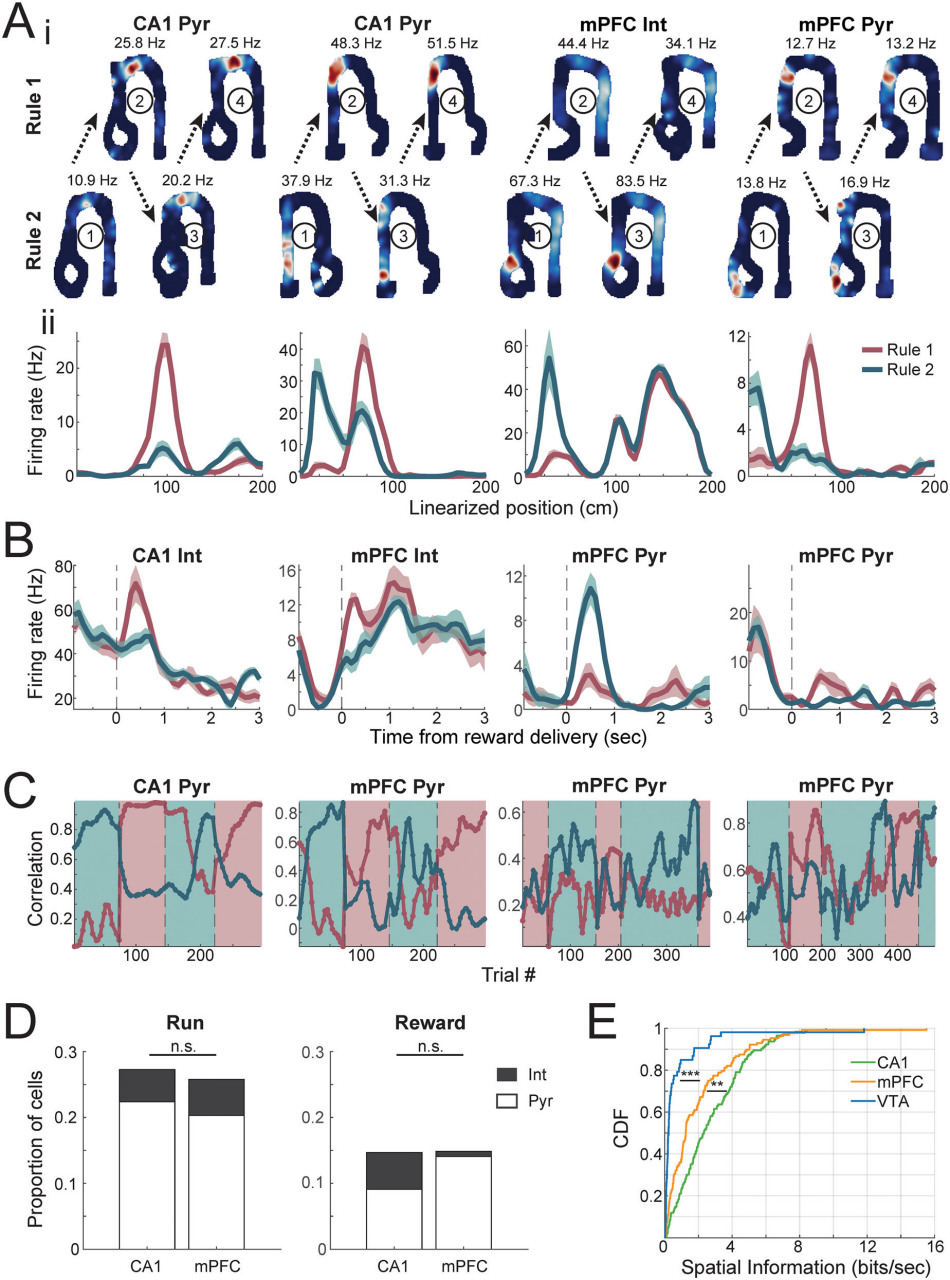
Remapping of single cells in CA1 and mPFC across rules. ***A***, Illustrative examples of spatial representations in CA1 and mPFC showing remapping on the common trajectories performed across the two rules, left to center or center to left. ***i***, 2D place fields at stable performance of Rule 1 (top) and Rule 2 (bottom) in interleaved blocks (first block for each rule showed on the left and second block shown on the right; numbers in circles denote the order of blocks), with peak firing rate on top. ***ii***, Linearized firing fields of the cells shown above (mean ± standard error). ***B***, Illustrative examples showing firing rate change of neurons at the same well locations across the two rules, aligned to reward onset for the same common trajectory across rules (left to center or center to left). ***C***, Correlation of single-cell firing patterns during individual trials to average firing patterns during stable performance of a given rule (>60th percentile of the performance of the day) during rule-switching blocks. Note the change in correlations that occur after rule switch. ***D***, Proportion of remapped cells for each region. During running: CA1 27.3% (39/143, 32 Pyr, 7 Int), mPFC 25.8% (33/128, 26 Pyr, 7 Int), *χ*^2 ^= 0.077, *p* = 0.78. At reward location: CA1 14.7% (21/143, 13 Pyr, 8 Int), mPFC 14.8% (19/128, 18 Pyr, 1 Int), *χ*^2 ^= 0.0013, *p* = 0.97. Fisher's exact test for proportions of remapped cells between regions. ***E***, Distribution of spatial information encoded by individual cells for each brain region. CA1, median 2.3 bits/s, *n* = 143; mPFC, median 1.3 bits/s, *n* = 128; VTA, median 0.23 bits/s, *n* = 53. CA1–mPFC *p* = 0.0010, mPFC–VTA *p* = 6.2 × 10^−8^, CA1–VTA *p* = 7.7 × 10^−17^, Kruskal–Wallis test with multiple-comparisons correction.

CA1 and mPFC neurons show spatially modulated firing activity (O’Keefe and Dostrovsky, 1971; O’Keefe, 1978; Hyman et al., 2010; Zielinski et al., 2019), and their features have also been implicated in rule and context representations (Wood et al., 2000; Eschenko and Mizumori, 2007; Griffin et al., 2007; Rich and Shapiro, 2009; Durstewitz et al., 2010; Ferbinteanu et al., 2011; Karlsson et al., 2012; Powell and Redish, 2016; Guise and Shapiro, 2017; Hasz and Redish, 2020). We therefore first asked the question if the firing activity of CA1 and mPFC neurons can reflect the current rule by showing rule-specific activity on trajectories. As the firing activity of VTA neurons showed much weaker spatial modulation (Fig. 2*E*), we focused only on CA1 and mPFC for this analysis. Single-unit remapping was investigated using firing activity on the two common trajectories that lead to reward for correct trials for both rules (“left to center,” inbound for Rule 1 and outbound for Rule 2, and “center to left,” outbound for Rule 1 and inbound for Rule 2), as lack of reward is known to destabilize firing activity on mazes (Krishnan et al., 2022). We found cells showing remapped activity across the two rules in both CA1 and mPFC regions (Fig. 2*A*; rules are run in alternating blocks with two blocks for each rule). Some cells show rate remapping (Fig. 2*A*, first example on left, CA1 excitatory cell), and some cells exhibited relocation of spatial firing to a different part of the track (CA1 and PFC excitatory cell examples). Additionally, reward-associated firing after the same physical trajectory but in different rule contexts also showed robust differences (examples with significant differences in Fig. 2*B*). These firing changes are not a result of recording drift over time, as similar patterns were observed both earlier and later in the day, with interleaved rule blocks. Furthermore, trial-wise cell firing vectors also exhibited increases in correlation with stable-performance firing vectors of the current rule as behavioral performance improved and became less similar (lower correlation) after the rule changed (Fig. 2*C*). Overall, we found that 27.3% of hippocampal CA1 cells and 25.8% of mPFC cells show significantly differentiated activity during running based on the current underlying rule and 14.7% CA1 and 14.8% mPFC neurons during reward. There was no significant difference in the ratio of rule-modulated neurons across regions (Fig. 2*D*).

### CA1 and mPFC ensemble representations distinguish the rules and show transitions during rule switching

**Figure 3.**
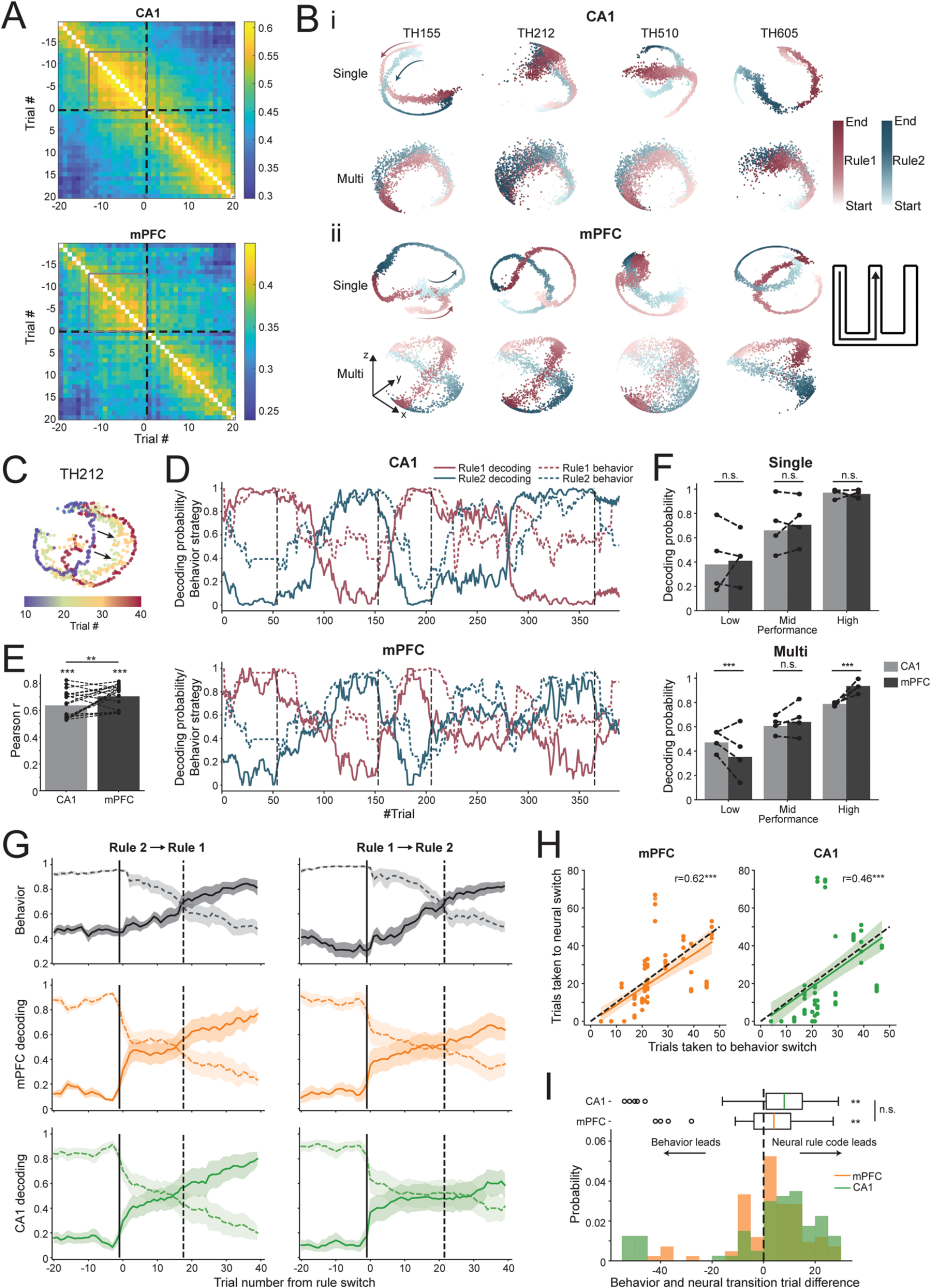
mPFC and CA1 ensembles encode rule representations and transitions. ***A***, Correlation of population activity vectors aligned to rule switch (dashed black line) in a trial-wise manner for CA1 and mPFC. Note the weakened stability shown by lower correlations following rule switches in both regions. Areas of stable correlations are denoted by gray squares. ***B***, CA1 and mPFC population activity show differential representations of the same trajectory across rules during good performance. Population activity space shown by dimensionality reduction using CEBRA (see Materials and Methods). A particular 3D view is illustrated, with arbitrary units for axes. ***i***, CA1 embeddings of the example common trajectory across rules (left to center, schematic on the right, with start and end denoting the beginning and end of the same trajectory across the two rules, respectively). Top, 3D embedding from models trained using individual animal's data. Bottom, Model trained with multisession CEBRA with data from all animals to capture common underlying structure across animals. ***ii***, Embeddings of the same trajectory but for mPFC. ***C***, An illustrative example showing that the mPFC population representation of the common trajectory shifts in the manifold space (shift denoted by arrow) during rule transition over the course of trials. Trajectory representations are grouped in 10 trials per color. ***D***, Decoding probability of each rule from an example animal shows CA1 (top) and mPFC (bottom) neural activity transitions from representing one rule to the other (Rule 1 in dark red, Rule 2 in teal) over a few to tens of trials after each rule switching (rule switching is denoted by dashed black vertical lines). Dashed colored curves show estimated behavior strategy similarity to the optimized behavior for each rule. ***E***, CA1 and mPFC rule decoding probabilities are highly correlated with behavior strategy. Mean Pearson’s *r*: between CA1 and behavior 0.64 ± 0.02, between mPFC and behavior 0.70 ± 0.02. Dots and dashed lines show the correlation between behavior and each decoding estimate (*n* = 20, 4 animals, 5 estimates each; see Materials and Methods). *P* values for all behavior–rule decoding correlations are <0.001. Behavior correlation with mPFC rule decoding probabilities was higher than with CA1 decoding (*p* = 0.0047, paired *t* test). ***F***, Rule decoding accuracy (proportion of trials decoded correctly) in each region, grouped by behavioral performance. Top, Decoding accuracy of single-animal models (low performance: CA1, 0.38, mPFC, 0.41, *p* = 0.33 for CA1 vs mPFC; mid performance: CA1, 0.66, mPFC, 0.71, *p* = 0.12; high performance: CA1, 0.97, mPFC, 0.96, *p* = 0.31, two-proportion *z* test). Bottom, Decoding accuracy using multi-animal models (low performance: CA1, 0.47, mPFC, 0.35, *p* = 1.3 × 10^−4^; mid performance: CA1, 0.61, mPFC, 0.64, *p* = 0.27; high performance: CA1, 0.79, mPFC, 0.93, *p* = 9.9 × 10^−12^). Dots and dashed lines show decoding accuracy for each animal under each condition (*n* = 4 animals). ***G***, Behavior strategy similarity (top), probabilities of mPFC rule decoding (middle), and CA1 rule decoding (bottom) aligned to rule-switching trials, shown separately as Rule 2 to Rule 1 (left) and Rule 1 to Rule 2 (right) switches. Behavior transitions occurred in 17 (median; IQR, 13–29) trials after the Rule 1 → Rule 2 switch, with mPFC and CA1 neural representations slightly leading behavior. For the switch type of Rule 2 → Rule 1, behavior started to reflect the new rule after 22 (median; IQR, 20.75–28.5) trials, with mPFC leading in contrast to CA1 lagging. ***H***, Correlation between the number of trials taken for behavior switch and neural representation switch, combined across rule switch types. Dashed lines indicate diagonal lines where the timing of neural transitions equals that of behavior switches. Left, mPFC and behavior, Pearson’s *r* = 0.62, *p* = 3.4 × 10^−10^. Right, CA1 and behavior, Pearson’s *r* = 0.46, *p* = 1.9 × 10^−5^. ***I***, Distribution of timing differences between behavior switch and neural representation switch. Behavior relative to mPFC transitions: median +4.0 (IQR, −3.75 to 10.5) trials, *n* = 80, *p* = 0.0044; behavior relative to CA1 transitions: median +8.0 (IQR, 1–15) trials, *n* = 78, *p* = 0.0040, Wilcoxon signed-rank test. For box plots, the boxes, whiskers, and circles indicate quartile, 1.5× IQR, and outliers, respectively.

We next investigated ensemble activity changes across rules in CA1 and mPFC and the dynamics of these changes in relationship to behavior. Trial-by-trial population vector similarity was computed and aligned to the first trial after rule switching, shown in the correlation plots in Figure 3*A*. Similar to previous research findings (Hasz and Redish, 2020), the firing pattern of both regions stabilized toward the end of running one rule (seen as increase in correlation with neighboring trials within a rule block) and quickly destabilized after changing to the other rule (Fig. 3*A*). We adopted a newly developed dimensionality reduction method CEBRA (Schneider et al., 2023), utilizing contrastive learning to visualize the difference of high-dimensional neural data across rules. For a common trajectory across the two rules (“left to center”), mPFC 3D embeddings using CEBRA qualitatively showed consistent separation across rules for all animals during stable performance (Fig. 3*Bii*, top row). These embeddings used training and test datasets within animals. We also used multisession training that sampled training data from all animals followed by testing within animals. The embeddings from this method revealed a similar structure across animals, implying animal-invariant features in the mPFC ensemble data for rule representations (Fig. 3*Bii*, bottom row). CA1 single-session embeddings showed similar separations between rules compared with mPFC. However, much weaker separations were observed in multianimal embeddings, suggesting a more unique latent space of CA1 rule-specific activity for individual animals (Fig. 3*Bi*). As animals switched from stable performance of one rule to another, we also observed a systematic transition in the manifold space (Fig. 3*C*, example mPFC manifold transitions over trials).

In order to quantify manifold changes across time, a kNN decoder was trained to decode the underlying rule using single-animal embeddings for each brain region (see Materials and Methods). Rule decoding probabilities using CA1 and mPFC data correlated highly with animals’ behavior strategy (Fig. 3*D*,*E*). Upon further inspection, behavior strategy showed a stronger correlation with mPFC rule decoding than with CA1 decoding (Fig. 3*E*). The decoding probability of the current rule increased with behavioral performance for both mPFC and CA1 (Fig. 3*F*, top; mean decoding accuracy for low performance, CA1 0.38, mPFC 0.41; mid performance, CA1 0.66, mPFC 0.71; and high performance, CA1 0.97, mPFC 0.96; performance thresholds are reported in Materials and Methods). However, when training the decoder using multi-animal embeddings, CA1 decoder performed much worse than mPFC, again implying more consistent rule coding across animals in mPFC and its stronger relevance to behavior than CA1 (Fig. 3*F*, bottom).

We further examined the timing between neural representation change and behavior strategy shift upon rule switch. The behavior and neural changes align with each other, with neural representations in both CA1 and mPFC transitioning more often before the behavior strategy than after (Fig. 3*H*,*I*), with no timing difference seen between CA1 and mPFC ensembles. On average, it took more trials to switch from Rule 1 to Rule 2, both in the neural representation and behaviorally (Fig. 3*G*), potentially due to asymmetric working memory components in Rule 2.

### VTA DA firing activity reflects and predicts reward outcomes

**Figure 4.**
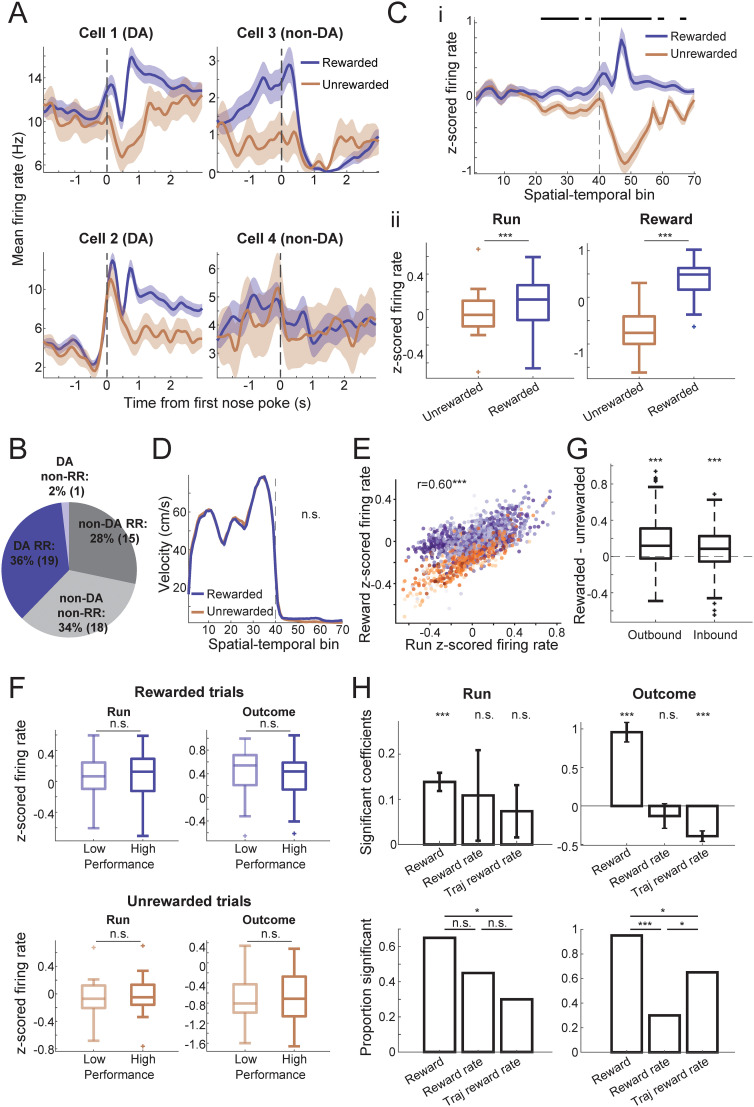
DA spiking activity carries reward-related information. ***A***, Illustrative examples showing that DA neuronal firing activity at reward wells signals robust differences between rewarded and unrewarded outcomes. Left, Example phototagged TH+ neurons. Right, Example non-DA neurons. Line, trial-averaged firing rate; shaded area, standard error. ***B***, Percentage of cell types and reward responses in VTA. RR, reward responsive at 200–1,200 ms after first nose pokes. DA RR 36% (19/53), DA non-RR 2% (1/53), non-DA RR 28% (15/53), non-DA non-RR 34% (18/53). ***C***, ***i***, Population response of putative DA neurons to reward outcomes during reward approach and at reward well. The bar on top indicates spatial/temporal bins with *p* < 0.05 after Bonferroni correction using Wilcoxon signed-rank tests. The vertical black dotted line is the first nose poke/reward at the reward well, also corresponding to the transition from spatial to temporal bins. Spatial bins during approach to reward well are from 1 to 40 (5–6 cm per bin, 200–240 cm in total), and temporal bins after arrival at the reward well are from 41 to 70 (100 ms per bin, 3 s in total). ***ii***, Average *z*-scored firing rates of all putative DA cells during rewarded versus unrewarded trials. Left, During run, starting from leaving the last reward/nose poke to the beginning of the current trial's reward/nose poke, rewarded median = 0.11, unrewarded median = −0.060, *p* = 1.8 × 10^−4^. Right, Reward time (200–1,200 ms after the first nose poke), rewarded median = 0.49, unrewarded median = −0.76, *p* = 5.6 × 10^−5^. Wilcoxon signed-rank test, *n* = 20 cells. ***D***, Control for running speed during trial. No significant differences are seen in running speed between rewarded and unrewarded trials (*p* = 0.12). ***E***, Strong correlation of DA population firing rates during reward approach running and at reward outcome (*r* = 0.60, *p* = 6.8 × 10^−148^). Colors from light to dark indicate performance from low to high. Orange, error trials; purple, correct trials. ***F***, DA firing rates show no difference between low- and high-performance periods. Top, Rewarded trials (median *z*-scored firing rate during running: low performance 0.065, high performance 0.13, *p* = 0.23; during reward outcome: low performance 0.54, high performance 0.44, *p* = 1.00). Bottom, Unrewarded trials (run: low performance −0.072, high performance −0.049, *p* = 0.23; outcome: low performance −0.81, high performance −0.71, *p* = 0.65; Wilcoxon paired rank test, *n* = 20 cells). ***G***, Difference in DA firing rates for rewarded versus unrewarded trials during running is present for both inbound trials (median difference 0.085, *p* = 1.8 × 10^−6^, *n* = 178 trials) and outbound trials (median difference 0.12, *p* = 1.0 × 10^−13^, *n* = 217 trials). ***H***, Multiple regression for DA neuron firing rates during run or reward outcome using the following parameters: current-trial reward, reward rate of the last five trials, reward rate of the same trajectory for the last five visits. Top, Mean significant coefficients. Current-trial reward: run: *β* = 0.14 ± 0.02 (mean ± SEM), *p* = 2.4 × 10^−4^; outcome: *β* = 0.95 ± 0.13, *p* = 1.6 × 10^−4^; reward rate: run: *β* = 0.11 ± 0.10, *p* = 0.36; outcome: *β* = −0.13 ± 0.16, *p* = 0.56; same-trajectory reward rate: run: *β* = 0.074 ± 0.058, *p* = 0.16; outcome: *β* = −0.39 ± 0.07, *p* = 2.4 × 10^−4^, Wilcoxon signed-rank test. Bottom, Proportions of DA neurons modulated by each factor. Run: current reward 65% versus reward rate 45%: *χ*^2 ^= 1.6, *p* = 0.20; current reward 65% versus same-trajectory reward rate 30%: *χ*^2 ^= 4.9, *p* = 0.027; reward rate 45% versus same-trajectory reward rate 30%: *χ*^2 ^= 0.96, *p* = 0.33. Outcome: current reward 95% versus reward rate 30%: *χ*^2 ^= 18.0, *p* = 2.2 × 10^−5^; current reward 95% versus same-trajectory reward rate 65%: *χ*^2 ^= 5.6, *p* = 0.018; reward rate 30% versus same-trajectory reward rate 65%: *χ*^2 ^= 4.9, *p* = 0.027. Fisher's exact test, *n* = 20 cells.

As midbrain DA plays a crucial role in reward signaling, we aimed to understand VTA DA spiking activity features during rapid rule switches guided by unexpected reward outcomes. We used an optotagging strategy in TH-Cre rats, which has been utilized in previous studies (Witten et al., 2011). Optotagging was performed during the first or last sleep session for each day of recording to not interfere with activity during task running. We recorded a total of 53 VTA neurons, out of which 20 phototagged cells were identified as putative DA neurons (with firing rates mean ± SD, 7.7 ± 3.0 Hz). Consistent with a vast body of literature, putative DA neurons showed increased firing rates when rewards became available, temporally aligned to the first poke at reward well, and decreased firing rates during reward omission (Fig. 4*A*; median *z*-scored firing rate for rewarded trials, 0.49, and unrewarded trials, −0.76; *p* = 5.6 × 10^−5^, Wilcoxon signed-rank test). Nontagged neurons exhibited a diverse range of activity, including 45.5% (15/33) of neurons responding to reward outcomes (Fig. 4*B*). Furthermore, we also observed that DA neurons had higher firing rates during running for trials that lead to rewards in comparison with unrewarded trials (Fig. 4*C*; median *z*-scored firing rate for rewarded trials, 0.11, and unrewarded trials, −0.060; *p* = 1.8 × 10^−4^). This difference became apparent and significant halfway through running on the trajectory, just after the choice point where the decision was made. Such observation is similar to previous findings of DA release in the striatum, and is likely related to reward expectancy/uncertainty (Howe et al., 2013). The running speed profile on the track was similar between rewarded and unrewarded trials, suggesting the DA firing rate difference is not caused by a change in motivation or vigor (Fig. 4*D*).

Based on the proposed link between DA spiking and reward prediction error, it is predicted that for rewarded trials, as behavioral performance improves, DA firing rates at the reward will decrease and DA firing rates during run will increase, and such trends would be the opposite for unrewarded trials (Watabe-Uchida et al., 2017). However, we observed a strong positive correlation between run and reward firing rates (*r* = 0.60, *p* = 6.8 × 10^−148^), and no obvious relationship between firing rates of either state to performance (Fig. 4*E*). We further tested this by separating the performance by trials into low- and high-performance halves, and found no difference in the firing rates across performance stages for either rewarded or unrewarded trials [*n* = 20 cells, median *z*-scored firing rate for rewarded trials (run, low performance 0.065, high performance 0.13, *p* = 0.23; outcome, low performance 0.54, high performance 0.44, *p* = 1.00) and unrewarded trials (run, low performance −0.072, high performance −0.049, *p* = 0.23; outcome, low performance −0.81, high performance −0.71, *p* = 0.65), Wilcoxon paired rank test, Fig. 4*F*].

In addition, we wanted to investigate if the DA reward prediction signal is present for working memory error as compared with rule/perseverative error that occurs after a rule switch. At each time of rule switching, two trajectories became unrewarding under all conditions, and two new ones became potentially rewarding when the sequence of arm visits was correct. For example, when Rule 1 changes to Rule 2, trajectories between the center and the right arms are no longer rewarding, which happens to be inbound error trials. Instead, left-to-right and right-to-left ones yield rewards. We classified the trials that animals ran on the newly unrewarding trajectories as perseverative errors (inbound errors), whereas the trials that animals made a wrong outbound choice for the current rule as working memory errors. If the DA firing activity only reflects a model-free system tracking overall reward probability, we would expect a difference only between perseverative errors and correct trials, but not for individual decisions dependent on working memory. We found that the prediction for reward outcome was present in DA firing regardless of the error type, even when comparing error trials to the nearest correct trials of the same outbound/inbound trajectory type (Fig. 4*G*, median *z*-scored firing rate difference between rewarded and unrewarded trials: outbound 0.12, *p* = 1.0 × 10^−13^, *n* = 217 trials; inbound 0.085, *p* = 1.8 × 10^−6^, *n* = 178 trials).

To summarize the influence of reward conditions and histories on DA firing rates, we fitted multiregression models for each cell's spiking rates during running and outcome periods, separately. The most significant factor was found to be the current trial's reward outcome (running: mean ± SEM coefficients 0.14 ± 0.02, significant for 65% of DA neurons; outcome: 0.95 ± 0.13, 95%), in comparison with a weaker impact of reward history (running: 0.11 ± 0.10, 45%; outcome: −0.13 ± 0.16, 30%) or same-trajectory reward history (running: 0.074 ± 0.058, 30%; outcome: −0.39 ± 0.07, 65%), for both running and reward states (Fig. 4*H*). Note that the current-trial reward outcome is a significant contributor to the approach run firing rate, and not just to the reward well firing rate that is expected.

### DA reward predicting feature develops after rule switching and coordinates with mPFC rule representation transition before behavior strategy adaptation

**Figure 5.**
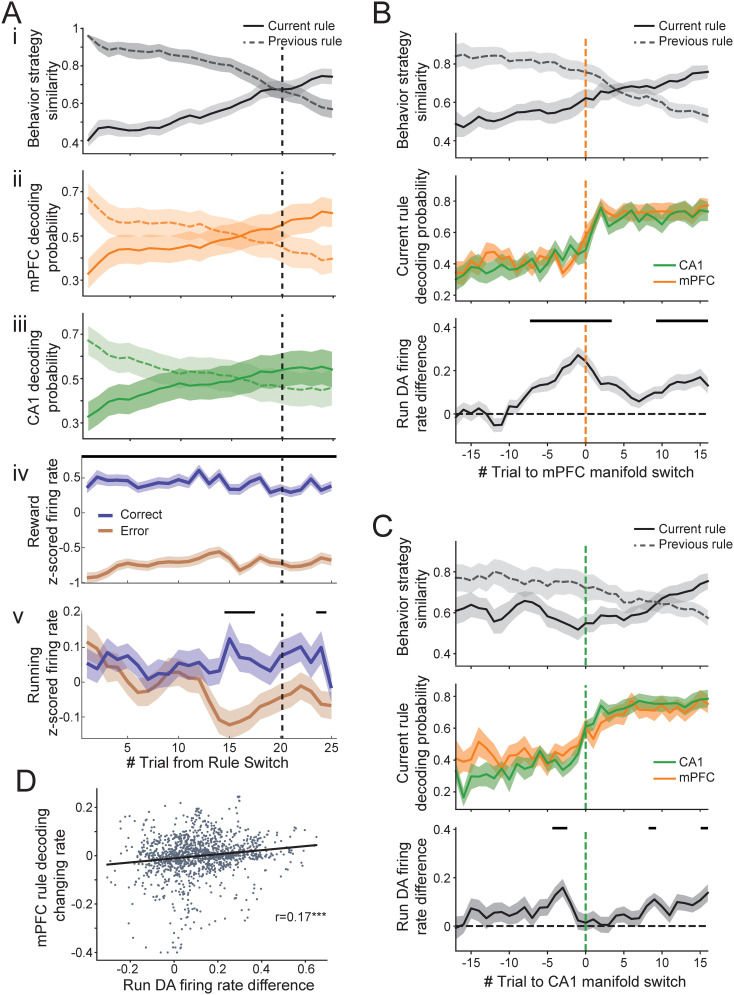
DA spiking activity relationship with neural manifold change and rule-switching behavior. ***A***, Behavior strategy (***i***), mPFC (***ii***), and CA1 (***iii***) decoding probability changes aligned to rule-switching trials. ***iv***, ***v***, Difference in DA neuron firing rates for rewarded versus unrewarded trials at well locations (***iv***) and during running (***v***), plotted as a function of trials aligned to the beginning of rule switch. Black bars indicate significant difference between rewarded and unrewarded trials with *p* < 0.05 after Bonferroni correction. The vertical black dotted line across subpanels denotes behavioral strategy switch trial. ***B***, Behavior strategy, CA1/mPFC decoding probability changes, and DA firing rate differences between rewarded and unrewarded trials, aligned to decoded rule representation switch in mPFC (vertical orange dotted line). Top, Behavior strategy similarity to optimized behavior of current and previous rules. Animals’ behavior adapted to the new rule on an average of four (IQR, −3.75 to 10.5) trials after mPFC showed correct rule decoding. Middle, CA1 and mPFC decoding accuracy of current rule. Bottom, *z*-scored DA firing rate difference for rewarded versus unrewarded trials during running. The black bars above indicate trials showing significant difference with *p* <0.05 after Bonferroni correction. ***C***, Same as ***B*** but aligned to CA1 rule representation switch (vertical green dotted line). ***D***, DA firing activity difference between rewarded and unrewarded trials is correlated with the changing rate of the mPFC manifold (Pearson’s correlation *r* = 0.16, *p* = 2.8 × 10^−9^, *n* = 1,361 trials).

Our findings in mPFC/CA1 rule coding and DA reward signals led us to the question of whether the firing rates of DA neurons predictive of reward outcomes develop as animals gather evidence of a rule change. Firing rates upon reward delivery were consistently higher than at reward omission (Fig. 5*Aiv*); however, the difference in DA neuron firing between rewarded and unrewarded trials during running only developed a few trials after the rule switch was implemented (Fig. 5*Av*) and a few trials prior to behavioral strategy switch (Fig. 5*Ai*). We further investigated whether this DA firing activity is coordinated with rule representation changes in CA1 and mPFC. When aligned to the trial where mPFC started to decode the current rule, DA firing rate difference during run between rewarded and unrewarded trials ramped up robustly toward this change point (Fig. 5*B*, bottom). The ramp up in DA firing rate difference was qualitatively more robust and consistent for mPFC representation switch rather than when aligned to CA1 representation switch (Fig. 5*C*, bottom). Animals’ behavior strategies adapted accordingly shortly after (on average four trials) the observed mPFC rule representation switch and DA prediction emergence (Fig. 5*B*, top). In addition, the DA firing activity difference between rewarded and unrewarded trials during maze running did not stay high as animals reached good, stable performance for the new rule. We observed a positive correlation between the DA firing rate difference and the rate of mPFC decoding probability change, which may suggest a gating mechanism of value/belief update (Fig. 5*D*). In summary, the reward predictive/expectancy property of DA firing during running is acquired post rule switch and is coordinated with the changes of mPFC rule representation and behavior strategy.

## Discussion

In the current study, we examined rule-related firing properties in the hippocampus and mPFC, VTA DA spiking activity during rule switching, and the temporal relationships between cognitive rule representation, reward prediction signal, and behavior adaptation. The mechanisms of rule representation and reward prediction have been generally examined separately, but it is vital to gain an integrated understanding of dynamics and temporal coordination between these mechanisms for supporting cognitive flexibility and behavioral adaptation. During rule switching, the presence or absence of rewards after specific actions can serve as feedback for organisms to reevaluate their behavior policy. A conflict between belief and outcome leads to a switch in internal rule representations, which can guide correct behavioral actions in the changed context. We therefore reasoned that there must exist some sort of temporal coordination between reward prediction and rule representation dynamics over the course of a few trials after rule switching, which will determine the time course of behavioral adjustments, thus allowing animals to overcome perseveration and adapt to the new context with appropriate actions. We found that mPFC and hippocampal CA1 population activity represented rule context. VTA DA firing activity responded to and predicted reward outcomes. The timings of the switches in mPFC rule representation and the emergence of correct reward prediction from VTA were coordinated. Furthermore, these changes in mPFC, CA1, and VTA DA firing activity led to the behavioral strategy transition, suggesting coordinated network activity supporting behavioral adaptation.

We implemented a spatial rule-switching task in a W/M maze, probing behavioral flexibility and working memory at the same time in the exact same environment. Rule 1 comprises a traditional W-maze alternation rule with the center arm as the home arm as in previous studies (Frank et al., 2000; Jadhav et al., 2012, 2016; Tang et al., 2017; Maharjan et al., 2018; Shin et al., 2019), and the home arm switched to the left arm for Rule 2, requiring the animals to change their strategy for optimal trajectory sequences across the rules. Notably, both rules incorporate a spatial working memory component (outbound component), requiring the animals to choose the opposite arm from the previous choice when embarking from the home arm. The return-to-home-arm trajectory is the inbound component with a spatial reference memory demand (Kim and Frank, 2009). The nature of the task assigned different memory demands of overlapping trajectories in the two rules, which allowed us to compare animal behavior, CA1/mPFC representations, and DA signaling for reward prediction during rule switch.

We first examined the differences in rule representation between the hippocampal CA1 and mPFC. Spiking activity changes associated with rule/context were observed in both brain regions (Guise and Shapiro, 2017; Hasz and Redish, 2020), with single neurons exhibiting remapping on the same trajectories across the two rule contexts. Examining ensemble coding using manifold analysis and decoding to current rule context revealed more robust and consistent coding of rule contexts across animals in mPFC, suggesting that similar neural representations may develop in mPFC to support separate rule contexts in the same physical trajectory space on mazes. Rule representations in mPFC and CA1 appeared to shift over the course of a few to tens of trials after rule switching, prior to observation of a behavioral strategy switch.

We observed more consistency across animals in mPFC ensemble decoding for rules, whereas CA1 ensemble representations were more animal-specific (Fig. 3*B*). In terms of timing, we did not see a significant difference between mPFC and CA1 when calculating the delay between neural transition times and behavioral transition times, although there was some variability across regions (Fig. 3*H*,*I*). However, the DA prediction differences that emerged after rule changes were more robustly aligned to mPFC transition times than to CA1 (Fig. 5*B*,*C*), and mPFC transition times were more closely aligned to behavioral strategy switch (Fig. 3*E*). Therefore, despite no significant transition timing differences between mPFC and CA1, our results suggest more relevance of mPFC to neural computations and behavior.

We observed that VTA DA neurons participated in reward prediction and this rule-specific feature developed quickly after rule switching. Previous literature has often focused on model-free RL reward prediction error; here, we instead tried to understand how midbrain DA neuronal firing activity changes during a rule-switching task that requires working memory. DA neuronal firing rates were highly correlated between running and reward outcome in a trial, which somewhat resembles previous findings in Coddington and Dudman (2018). In addition, we did not find a correlation between DA spiking activity and performance at the population level. These results contrast with the prediction based on classical conditioning that firing activity increases toward cue/beginning of a trial and slowly diminishes upon reward delivery (Schultz et al., 1997; Fiorillo et al., 2003; Amo et al., 2022). Rather, our findings suggest that DA signaling may incorporate rule-based and working memory-dependent prediction information, potentially from cortical input.

We found a delay in animals’ behavioral strategy adjustment in comparison with rule representation and reward prediction. Animals continued to sample from wrong trajectories, although at a lower frequency, after manifolds shifted to the new rule and DA signal could predict the reward outcome. This can be explained by simply reserving a small chance for random exploration or perhaps by the theory of active inference (Friston et al., 2012, 2014). In this theory, the dopamine signal minimizes surprise instead of cost. Therefore, sampling from extra trials could help confirm animals’ beliefs if correct and eventually stabilize behavioral sequences.

How is information processed in these regions? Does VTA receive reward, expectation, or reward prediction error (RPE) information from other regions? Lisman and Grace (2005) suggested that novelty information is conveyed to VTA from the hippocampal formation through an indirect route, possibly with inhibitory afferents from the accumbens and ventral pallidum. In return, DA fibers innervate the dorsal hippocampus and thus enhance LTP (Huang and Kandel, 1995; Otmakhova and Lisman, 1996; Li et al., 2003; Rosen et al., 2015; Sayegh et al., 2024). In terms of DA–cortical interactions, VTA receives abundant prefrontal glutamatergic projections (Geisler et al., 2007), and their impact on reward signaling is multifaceted. Input from frontal cortices is critical for conveying the predictive and incentive features of a cue (Pan et al., 2021). PFC conveys belief state information to the dopaminergic system and affects RPE computation when hidden states are involved (Babayan et al., 2018; Starkweather et al., 2018). Additionally, Amo et al. (2024) showed glutamatergic input to VTA already carried RPE signal, challenging the classic view of local computation of RPE in VTA by combining different aspects of reward and expectation from glutamatergic and GABAergic inputs (Kawato and Samejima, 2007; Keiflin and Janak, 2015). An intriguing future direction of research could focus on dissecting computational features or components of RPE in these brain regions involved in the reward circuit, particularly when multiple cognitive demands are present.

It is important to differentiate between DA firing activity and DA release in downstream areas. We did not observe an obvious increase in DA spiking activity during the goal approach. However, downstream release in the striatum has been reported to ramp up toward goal (Howe et al., 2013; Hamid et al., 2016), suggesting partially dissociated activity potentially caused by local modulation (Mohebi et al., 2019). It would be interesting to examine if the working memory-dependent reward prediction signal is present in the DA release profile as in VTA DA spiking activity, and if so, how that influences its downstream striatal and cortical areas.

The exact mechanism of information transfer and cooperation requires further investigation. For example, Fujisawa and Buzsáki (2011) proposed a 4 Hz rhythm orchestrating neuronal activity in the hippocampus, PFC, and VTA during a similar working memory task. DA administration in PFC increased HPC–PFC theta coherence and PFC phase locking to CA1 theta (Benchenane et al., 2010). PFC theta sequences selectively coordinate with CA1 theta sequences depicting future trajectory choices (Tang et al., 2021). Therefore, altered DA neuron spiking activity might impact or be impacted by the online processes of action evaluation and selection during running. Another possible mechanism is through coordinated reactivation/replay. It has been shown that hippocampal reactivation is associated with increased firing activity of VTA cells as a potential mechanism for memory consolidation (Gomperts et al., 2015). CA1–PFC coordinated replay is biased toward behavioral choice and therefore can potentially participate in working memory and decision-making (Shin et al., 2019). This hippocampocortical/subcortical temporal coordination during offline states may serve as substrates for value updates, memory consolidation, action evaluation, and planning. Our study focused on the behavioral time scale, but investigating simultaneous activity on a shorter time scale (50–200 ms) can potentially uncover detailed mechanisms of interregional communication.

## Conclusion

To understand the temporal coordination between cognitive systems and reward systems, we recorded from the rat hippocampal CA1, mPFC, and VTA during a rapid rule-switching task. CA1 and mPFC exhibited rule representation in their population activity. DA neuron firing activity in VTA gradually became predictive of reward outcomes after rule switching, and this predictive feature developed together with the mPFC representation transition to reflect the new rule. These neural representations changed in advance of behavioral adaptation by a few trials. Together, our study revealed a synchronized update of information across mPFC, HPC, and VTA that potentially supports behavioral flexibility.
